# Editorial

**DOI:** 10.1007/s11943-021-00291-2

**Published:** 2021-09-03

**Authors:** Timo Schmid, Markus Zwick

**Affiliations:** 1 Lehrstuhl für Statistik und Ökonometrie, Otto-Friedrich-Universität Bamberg, Bamberg, Deutschland; 2 Institut für Forschung und Entwicklung in der Bundesstatistik, Statistisches Bundesamt, Gustav-Stresemann-Ring 11, 65189 Wiesbaden, Deutschland

Liebe Leserinnen und Leser des AStA Wirtschafts- und Sozialstatistisches Archiv,

wir freuen uns, Ihnen die zweite Ausgabe des AStA Wirtschafts- und Sozialstatistisches Archiv (WiSoStA) des Jahres 2021 vorlegen zu können. Es ist auch dieses Mal gelungen, Ihnen ein breites Themenspektrum anzubieten. Wir verstehen das WiSoStA als eine wissenschaftliche Zeitschrift, in der insbesondere auch die methodologische Betrachtung der Statistik ihren Platz hat. Zwei der Beiträge in diesem Heft zeigen das sehr deutlich. Statistik als Basis um ‚Realitäten‘ abzubilden war und ist politisch relevant. ‚Realitäten‘ sollten für eine evidenzbasierte Politik Ausgangspunkt der Entscheidung sein. Diese sind aber häufig nicht rein objektiv, sondern hängen auch von der Sichtweise des Betrachtenden ab. Das gilt im Besonderen in unterschiedlich ausgeprägten Gesellschaft- und Wirtschaftssystemen.

In demokratisch verfassten Staaten ist die Politik ein wichtiger Partner für die jeweiligen statistischen Institutionen. Die Politik schafft die gesetzlichen Grundlagen, damit es der amtlichen Statistik gelingen kann, eine gegenwärtig durch den rasanten digitalen Wandel geprägte ‚Realität‘ adäquat abzubilden. Dies zeigt der Beitrag von Bork et al. (2021). Die Autoren tragen im Bundesministerium für Wirtschaft und Energie (BMWi) Verantwortung für die Weiterentwicklung der Wirtschaftsstatistiken. In ihrem Beitrag ‚Wirtschaftsstatistik im Wandel‘ legen sie dar, wie das BMWi in der zu Ende gehenden Legislaturperiode gemeinsam mit den Statistischen Ämtern des Bundes und der Länder umfassende Reformen der bestehenden Statistikgesetzgebung angestoßen haben. Dies war insbesondere in den Themenbereichen Basisregister für Unternehmensdaten, Verwaltungsdaten-Informationsplattform sowie im Rahmen der Reform des Preisstatistikgesetzes der Fall. Im Kontext der Umsetzung des EU-Unternehmensbegriffs zeigt sich, wie sehr die konzeptionelle statistische Arbeit unsere Wahrnehmung von Realität beeinflusst. Der administrativ geprägte deutsche Unternehmensbegriff wird nun durch einen EU-weit harmonisierten Unternehmensbegriff ersetzt. Dadurch verändert sich die Anzahl der Unternehmen in Deutschland, ohne dass sich real etwas an den Märkten verändert hätte. Der neue EU-Unternehmensbegriff scheint konzeptionell durchaus geeigneter. Der Nachweis, dass dieser auch adäquat erfasst werden kann, steht noch aus, siehe Beck et al. (2020).

Im Kontrast zu einer partnerschaftlichen Zusammenarbeit von Statistik und Politik steht der Beitrag ‚Statistik im Sozialismus‘ von Walter Krämer und Klaus Leciejewski (2021). In den Ausführungen zeigt sich, dass Statistik sowohl in der Historie als auch gegenwärtig immer wieder als steuerndes Element der Politik genutzt wurde. Wie von der Lippe (1996) für die DDR darlegt, kann Statistik auch für eine zielgerichtete Fehlinformationspolitik genutzt werden. Häufig ist ein Problem hierbei, dass mit dem Aufbau eines statistischen Wunschbildes von Gesellschaft und Wirtschaft, die tatsächlichen Realitäten seitens der Entscheidungstragenden immer weniger wahrgenommen werden (können). Zum einen glaubt man selbst gerne an diese Wunschbilder, zum anderen ist es kaum möglich, gleichzeitig sowohl eine politisch gewünschte als auch eine adäquate Wirklichkeit statistisch abzubilden. Eine Statistik beeinflussende Politik beraubt sich selbst ihrer sachgerechten Entscheidungsgrundlagen. Ein Mittel gegen ‚Fake News‘ von staatlicher wie privater Seite ist eine statistisch gebildete Bevölkerung. Dieses Themengebiet wird heute unter dem Begriff ‚Statistical Literacy‘ beziehungsweise ‚Data Literacy‘ diskutiert. Ein Sonderheft im WiSoStA gibt hierzu einen Überblick (Krämer et al. 2019).

Wie Visualisierungen auch komplexere Themen intuitiv erfassbar machen können, zeigen Rendtel, Neudecker und Fuchs (2021) in ihrem Beitrag ‚Ein neues Web-basiertes Verfahren zur Darstellung der Corona-Inzidenzen in Raum und Zeit‘. Die vorgestellte Web-Applikation benutzt ein alternatives statistisches Konzept zur Darstellung von Corona-Inzidenzen. Statt der üblichen Annahme einer Gleichverteilung über einer Referenzfläche schätzt das zugrundeliegende Verfahren, die Verteilung im Raum über einen Simulated EM-Algorithmus. Diese werden dann in einem nächsten Schritt visualisiert.

Mit den Auswirkungen der Corona-Krise beschäftigt sich auch der Aufsatz von Maier, Mönnig und Zika (2021). Die Autorin und Autoren wenden das sogenannte Modellsystem QINFORGE auf mögliche wirtschaftlichen Auswirkungen der COVID-19-Krise an. Das Modellsystem setzt sich aus einer Bevölkerungsprojektion, einem Bildungsmodell einem makroökonometrischen Input-Output-Modell zusammen. Die Ergebnisse des Modells zeigen, dass durch die Krise vor allem Berufe in der Gastronomie, dem Tourismus sowie der Kunst und Kultur betroffen sind.

Im Interview erklärt Manfred Deistler, welchen Charme die Ökonometrie besitzt. Das Gespräch mit dem international renommierten Ökonometrie- und Zeitreihenanalysten führte Walter Krämer (2021) im vergangenen Jahr am Rande der Tagung des ökonometrischen Ausschusses des Vereins für Socialpolitik auf Schloss Rauischholzhausen bei Marburg.

Nun wünschen wir Ihnen, liebe Leserinnen und Leser, viel Spaß bei der Lektüre der zweiten Ausgabe 2021 des AStA Wirtschafts- und Sozialstatistisches Archiv.

Timo Schmid und Markus Zwick
